# Metabolome and Transcriptome Analyses of the Molecular Mechanism Underlying Light-Induced Anthocyanin Accumulation in Pepper (*Capsicum annuum* L.) Peel

**DOI:** 10.3390/cimb47090774

**Published:** 2025-09-18

**Authors:** Qinqin He, Liming He, Zongqin Feng, Yunyi Xiao, Qiucheng Qiu, Jiefeng Liu, Hanbing Han, Xinmin Huang

**Affiliations:** 1 Guangdong Provincial Key Laboratory for Green Agricultural Production and Intelligent Equipment, College of Biology and Food Engineering, Guangdong University of Petrochemical Technology, Maoming 525000, China; xiaohe1507@126.com (Q.H.); heliming7799@163.com (L.H.); zongqinfeng@163.com (Z.F.); xyunyi@126.com (Y.X.); gdmmljf@126.com (J.L.); hhb05@126.com (H.H.); 2 Maoming Agricultural Science and Technology Promotion Center, Maoming 525000, China; mszyqqc@126.com

**Keywords:** pepper, anthocyanin biosynthesis, light, metabolic network, transcription factor

## Abstract

Under light exposure, certain pepper cultivars synthesize large amounts of anthocyanins in their pericarps, with the illuminated areas exhibiting black coloration. However, research on light-induced anthocyanin formation in pepper fruit, particularly the related metabolites and genetic changes, remains limited. To identify the key genes involved in localized anthocyanin synthesis under light conditions, we investigated the black pericarps (light-exposed) and green pericarps of pepper variety MSCJ1 under illumination. Metabolomics analysis identified 579 metabolites in the black and green pepper pericarps, with 50 differentially accumulated metabolites. Petunidin-3-(6″-*p*-coumaroyl-glucoside) and delphinidin-3-*p-*coumaroyl-rutinoid accumulation represented the main factor underlying light-induced blackening of the pericarp. RNA-seq identified 121 differentially expressed genes that were significantly enriched in the flavonoid biosynthesis pathway. The genes for phenylalanine ammonia lyase (*Capana09g002200*, *Capna09g002190*), cinnamic acid hydroxylase (*Capana06g000273*), chalcone synthase (*Capana05g002274*), flavonoid 3-hydroxylase (*Capana02g002586*), flavonoid 3′-hydroxylase (*MSTRG.15987*), dihydroflavonol 4-reductase (*Capana02g002763*), anthocyanin synthase (*Capana01g000365*), UDP glucosyltransferase (*Capana03g000135*), and glutathione S-transferase (*Capana02g002285*) were key genes for anthocyanin synthesis and transport. Transcription factors bHLH (*Capana09g001426*, *Capana09g001427*), HSFB3 (*Capana05g000086*), and TCP4 (*Capana07g002142*) participated in the regulation of anthocyanin synthesis. These results broaden our understanding of the mechanism of light-induced anthocyanin synthesis in pepper peel.

## 1. Introduction

Pepper (*Capsicum annuum* L.) is an important economic crop in the Solanaceae family that originated in the Americas. With its vibrant colors and unique flavor, pepper has become an indispensable fresh vegetable and condiment in human diets [1,2]. Pepper contains antioxidants, such as capsaicin, vitamins, carotenoids, phenols, and flavonoids, which can effectively remove reactive oxygen species (ROS) and protect cell structures from free radical damage caused by oxidative processes; moreover, these substances have anti-inflammatory effects, stimulate the immune system, and reduce the risk of chronic diseases [3,4,5,6]. For example, capsaicin can inhibit the proliferation of breast cancer cells and promote the apoptosis of cancer cells by targeting FBI-1 [7]. It can also induce autophagy in renal cell carcinoma through the AMP-activated protein kinase and mechanistic target of rapamycin complex 1 pathway, thereby playing a therapeutic role in renal cell carcinoma [8]. Capsanthin extracted from red peppers can be used in the cosmetics industry as a raw material for makeup production [9,10]. In addition, colorful ornamental chili peppers have become popular in landscaping and home gardening [11,12]. Due to the widespread use of chili in the food, pharmaceutical, and cosmetics industries, the demand for chili worldwide continues to rise. According to FAO statistics, the global chili planting area reached 2,078,042 ha in 2024, mainly in Brazil, India, China, and Mexico, with a total output of 39 million tons.

Fruit color is an important characteristic of pepper because the pigments that form the color are related to the nutrition and flavor of chili peppers and confer special health benefits, such as beneficial antioxidant effects and cancer prevention activity [3,13,14]. New varieties of pepper with different fruit colors, such as green, red, yellow, white, purple, and black, have been cultivated [15]. The pigments in green fruits mainly include chlorophyll [16], those in red and yellow chili fruits are carotenoids [17], and those in purple and black fruits are anthocyanins and chlorophyll, with higher anthocyanin contents corresponding to a darker color [15]. White fruits do not contain pigments, such as chlorophyll and carotenoids [18].

Anthocyanins are a class of water-soluble natural pigments widely present in plants, and they are also flavonoid compounds, which mainly include six categories: cyanidin, pelargonidin, peonidin, delphinidin, petunidin, and malvidin [19]. In plant cell vacuoles, various colors, such as red, purple, and blue, appear with changes in pH [20]. Anthocyanins not only endow plants with rich colors but also attract pollinators and frugivores; thus, they play an important role in plant reproduction [21,22,23,24]. In addition, anthocyanins enhance the photostability of the seedling photosynthetic system, absorb ultraviolet radiation, remove ROS, reduce chlorophyll photobleaching under strong light, maintain osmotic balance, and improve plant stress resistance [25,26,27,28]. Modern medical research shows that anthocyanins, as powerful natural antioxidants, can efficiently eliminate free radicals in the body, significantly reduce oxidative stress damage to cells, and inhibit the release of inflammatory factors; moreover, these compounds exhibit multiple effects in cardiovascular protection, such as reducing blood lipids, improving vascular endothelial function, and preventing atherosclerosis, and show broad medical application prospects in anti-aging, vision protection, anti-cancer, and other fields [29,30,31,32,33]. As the skin of the edible parts of purple and black peppers are rich in anthocyanins, which can improve human health, these vegetables are becoming increasingly popular in the consumer market [34,35].

Anthocyanins are synthesized in the cytoplasm of plant cells, starting from the phenylalanine metabolic pathway, and catalyzed by phenylalanine ammonia lyase (PAL), cinnamic acid hydroxylase (C4H), and coumarin CoA ligase (4CL) to form *p-*coumaroyl CoA. Under the catalysis of chalcone synthase (CHS) and chalcone isomerase (CHI), coumarin CoA forms naringin. Subsequently, naringin can form anthocyanins under the catalysis of flavonoid 3-hydroxylase (F3H), flavonoid 3′-hydroxylase (F3′H), flavonoid 3′-hydroxylase (F3,5′H), dihydroflavonol 4-reductase (DFR), and anthocyanin synthase (ANS) [36,37]. Anthocyanins undergo further glycosylation modification by UDP glucosyltransferase (UGT) and are ultimately transferred to vacuoles for storage by glutathione S-transferase (GSTF) [38,39].

With the increasing demand of consumers for chili peppers rich in anthocyanins, breeding purple chili varieties has become one of the main directions for chili variety breeding. In addition, studying the regulation mechanism of anthocyanin synthesis in chili peppers is the basis for breeding new varieties. The anthocyanins in the fruits of the chili varieties Árbol and Uvilla are mainly composed of the glycoside delphinidin, and the highest content is observed at 20 days after flowering. The anthocyanin content in the purple-skinned Uvilla variety is higher than that in Árbol. The expression levels of the *F3′5′H*, *DFR*, *UFGT*, and *GST* genes are positively correlated with anthocyanin accumulation [40]. Meng et al. found that delphin chloride is the main cause of fruit color formation in two varieties of purple peppers (L66 and L29). The expression levels of the *PAL*, *C4H*, *CHI*, *DFR*, *ANS*, and *UFGT* genes in the fruits of purple varieties are higher than those in the fruits of green varieties (L9) [35]. HPLC-MS analysis showed that the purple pepper variety Co62 contains two types of cyanide-based anthocyanins. Further analysis revealed that *F3′H* and *F3′5′H* are key enzyme genes involved in the formation of two anthocyanins. *CaANT1*, *CaANT2*, *CaAN1*, and *CaTTG1* can activate anthocyanin accumulation by forming e MYB-MYB-bHLH-WD40 transcription complexes [34]. Light is also an important factor affecting anthocyanin synthesis. Light exposure increases the anthocyanin content in the peel of the purple pepper variety HNUCA21, while shading inhibits anthocyanin synthesis. Weighted gene co expression network analysis showed that *CHS*, *DFR*, *CHI*, *EGL1*, two *MATE*, and two *WRKY44* genes are involved in anthocyanin synthesis and their expression is upregulated by light [41]. UV-B irradiation can cause the epidermis of pepper variety 19Q6100 to turn purple. Yeast one hybrid and yeast two hybrid experiments found that CaMYB113 interacts with CabHLH143 and CaHY5 and participates in UV-B-induced anthocyanin biosynthesis in pepper fruit by regulating the expression of key enzyme genes involved in anthocyanin synthesis [42].

Although progress has been made in identifying the key genes related to anthocyanin composition and synthesis in purple or black peppers, most studies have focused on different color varieties or different developmental stages. Moreover, research on the metabolites and genetic changes of light-induced anthocyanin formation in pepper fruit is relatively limited. Therefore, we used the MSCJ1 variety as the material because it accumulates anthocyanins when exposed to natural sunlight, resulting in a black color, while the shaded area remains green. By identifying and analyzing the metabolic change landscape and potential metabolic regulatory network between black and green fruit peels through metabolomics and transcriptome analysis, this study can deepen our understanding of the regulatory mechanism underlying light-induced changes in anthocyanin biosynthesis in pepper. Moreover, the findings may provide new target genes or screening markers for the breeding of new varieties of high-anthocyanin pepper.

## 2. Materials and Methods

### 2.1. Plant Materials

The pepper variety MSCJ1 was obtained from Maoming Maoshu Seed Industry Technology Co., Ltd. (Maoming, China), and planted in the experimental field of Guangdong University of Petrochemical Technology under conventional cultivation management. Black (B) and green (G) parts of the pepper peel were collected 20 days after flowering, and 30 fruits were mixed into one sample. The samples were then frozen with liquid nitrogen and stored in an ultra-low temperature refrigerator until required for metabolomics, transcriptome, and qPCR analyses. Three biological replicates were performed per sample.

### 2.2. Metabolite Detection and Analysis

Vacuum freeze-drying was performed on the pepper samples, followed by grinding. Subsequently, 100 mg of the powdered sample was weighed, and metabolites were extracted using 1.0 mL of 70% methanol containing 0.1 mg/L lidocaine as an internal standard at 4 °C for 16 h. The sample was vortexed every 4 h for more thorough extraction. The extraction solution was centrifuged at 10,000× *g* for 10 min, and the supernatant was filtered using a microporous membrane (0.22 μm pore size). The filtrate was detected for metabolites using an API 6500 QTRAP LC/MS/MS (AB Sciex, Framingham, MA, USA) system. Quality control (QC) samples were prepared by mixing sample extracts. Raw data obtained from mass spectrometry analysis were processed, and metabolites were qualitatively analyzed based on public and self-built databases. Principal component analysis (PCA) and orthogonal partial least squares–discriminant analysis (OPLS-DA) analyses were performed for the metabolite data. The variable importance in projection (VIP) score of the OPLS model measures the contribution of each metabolite to the model. Metabolites with VIP ≥ 1 and *p* < 0.05 (*T* test) are considered differentially accumulated metabolites (DAMs) between two samples [43]. Metabolite detection and analysis were conducted at Gene Denovo Biotechnology Co. (Guangzhou, China).

### 2.3. RNA Sequencing

Total RNA was extracted from pepper fruits using the TRIzol reagent kit (Invitrogen, Carlsbad, CA, USA), and evaluated by agarose gel electrophoresis, Agilent 2100 bioanalyzer (Agilent Technologies, Palo Alto, CA, USA), and Qubit2.0 Fluorometer (Invitrogen, Carlsbad, CA, USA) for RNA integrity, purity, and concentration, respectively. Qualified samples were subjected to a Ribo-Zero^TM^ Magnetic Kit (Epicenter, Madison, WI, USA) to remove rRNA and enrich mRNA. Using ultrasound to interrupt mRNA, the first strand of cDNA was synthesized using fragmented mRNA as a template. Subsequently, the RNA strand was degraded using RNaseH, and the second strand of cDNA was synthesized in the DNA polymerase I system. After purification, the double stranded cDNA was subjected to terminal repair, followed by the addition of an A tail, and the connection of sequencing adapters. Approximately 200 bp of cDNA was screened for PCR amplification to construct a sequencing library. Sequencing was performed using an Illumina HiSeq2500 (San Diego, CA, USA) at Gene Denovo Biotechnology Co. (Guangzhou, China). Offline data were filtered to obtain clean reads, and bowtie2 (version 2.2.8) was used to remove reads aligned with ribosomal RNA. Reads that were filtered out of the ribosomal RNA were aligned to the chili genome solgenomics_C. annuum_zunla using HISAT 2 (version 2.2.4) [44].

### 2.4. Differentially Expressed Gene Screening and Enrichment Analysis

Fragments per kilobase of transcript per million mapped reads method was used to calculate gene expression levels. OmicSmart (https://www.omicsmart.com/, accessed on 15 October 2024) was used to analyze the Pearson correlation coefficients between six samples [45]. DESeq2 (version 1.30.0) was used to screen differentially expressed genes (DEGs) in different-colored pepper peels with false detection rate (FDR) <0.05 and|log2-fold change (FC)| > 1 [46]. After obtaining the DEGs, GO functional analysis and KEGG pathway analysis were performed on the DEGs [47,48]. Gene set enrichment analysis (GSEA) was performed using GSEA software (version 4.2.3) and the MSigDB database, and significantly enriched gene sets between the two different-colored fruit peels were identified based on normalized enrichment scores > 1, *p* < 0.05, and FDR < 0.25 [49].

### 2.5. Association Analysis of Key Metabolites and Genes Involved in Anthocyanin Synthesis

The regulatory mechanism of anthocyanin synthesis in chili can be better clarified by conducting correlation analyses on metabolites and genes that regulate anthocyanin synthesis. Differential metabolites, key enzyme genes, and transcription factors in the anthocyanin synthesis pathways were screened based on KEGG pathway analyses, and the correlation between them was assessed using Pearson correlation coefficients. Heatmaps and correlation analyses were performed using OmicSmart (https://www.omicsmart.com/, accessed on 10 July 2025) [45].

### 2.6. Quantitative Reverse Transcription PCR (qRT-PCR)

Twelve DEGs were randomly selected, and specific qRT-PCR primers were designed based on genomic data (Supplementary Table S1). Following the qRT-PCR method of Huang et al., *CaActin* was used as the internal reference gene, and the relative gene expression level was calculated using the 2^−ΔΔCT^ method [50]. Three biological replicates were performed for the qRT-PCR analysis, and the results are displayed as the mean ± SE. SigmaPlot v.11 (Systat, San Jose, CA, USA) was used for data statistical analysis and mapping.

## 3. Results

### 3.1. Global Metabolic Characteristics of Different-Colored Pepper Peels

**Figure 1:**
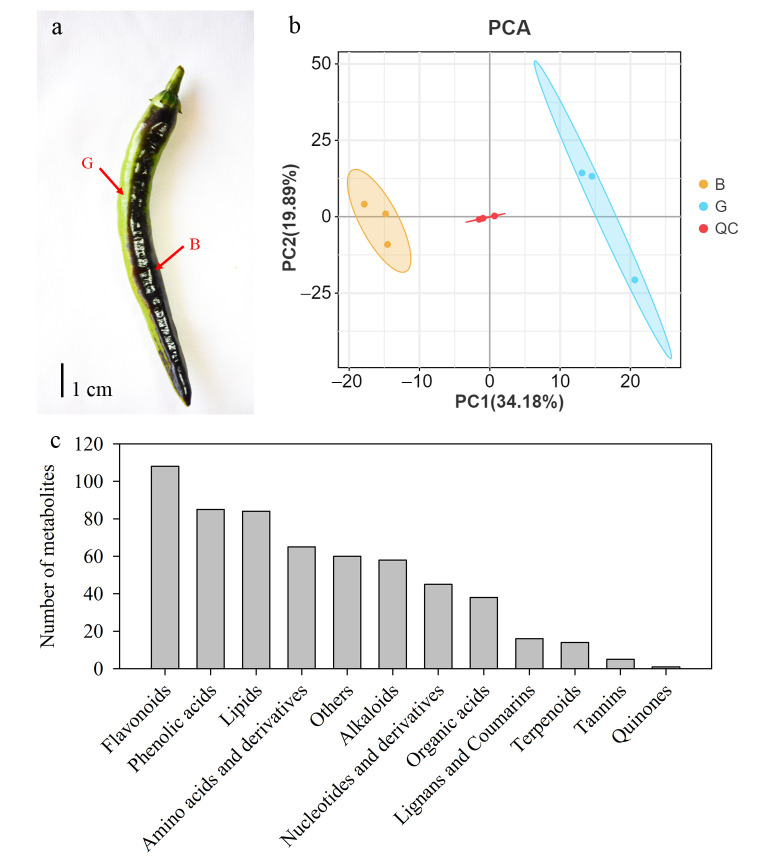
Summary of the metabolome of pepper (*Capsicum annuum* L.) with different-colored tissues. (**a**) Light-induced differential accumulation of pigment in pepper fruits, light area (black fruit peel, B), and backlit area (green fruit peel, G); (**b**) Principal component analysis (PCA) score plot of different color samples and quality control (QC) samples; (**c**) Metabolites in different-colored tissues of pepper fruits.

When the chili variety MSCJ1 is planted in the field, the area of the fruit exposed to light accumulates a large amount of anthocyanins and appears black, while the backlit surface shows no anthocyanin accumulation and appears green (Figure 1a, Figure S1). Metabolite detection was performed on the black and green areas of the fruit peel, and the total ion chromatogram (TIC) of the mass spectrometry analysis of different QC samples revealed overlapping results between the positive ion TIC (Figure S2a) and negative ion TIC (Figure S2b). This finding indicated that the metabolite extraction and detection results were accurate and reliable. PCA analysis was conducted on the metabolomics data from the two different-colored fruit peels. The results showed that three replicates of black fruit peel (B) clustered on the negative side of PC1 and three replicates of green fruit peel (G) clustered on the positive side of PC1, indicating good repeatability among the three samples of the same color. However, differences in metabolites were observed among the different-colored pepper fruit peels (Figure 1b). A total of 579 metabolites were identified from the two colored pepper peels, and they could be divided into 12 categories: flavonoids (108), phenolic acids (85), lipids (84), amino acids and derivatives (65), others (60), alkaloids (58), nucleotides and derivatives (45), organic acids (38), lignans and coumarins (16), terpenoids (14), tannins (5), and quinones (1) (Figure 1c). Among them, there were eight types of anthocyanins, including four types of cyanidin anthocyanins, two types of delphinidin anthocyanins, and two types of morning glory anthocyanins (Supplementary Table S2).

### 3.2. DAMs Between Two Different-Colored Pepper Peels

**Figure 2:**
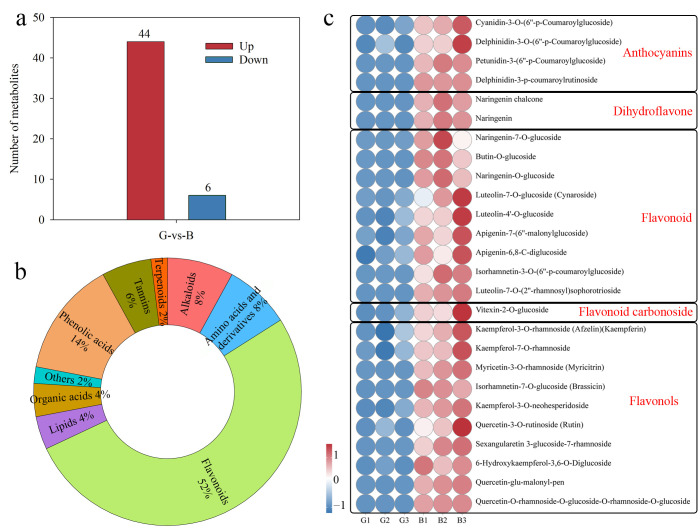
Differentially accumulated metabolites of pepper (*Capsicum annuum* L.) with different-colored tissues. (**a**) Number of differential accumulated metabolites; (**b**) Differential accumulated metabolites classification; (**c**) Heat map of the flavonoid content. The heatmap displays Z-score-normalized metabolite content. B: black fruit peel, G: green fruit peel.

OPLS-DA was used to perform multivariate statistical analyses on the metabolites of the two different colors of pepper skin and establish a model. The results showed that the R2Y of the obtained model was 0.999 and Q2Y was 0.98, indicating the sufficient capture of metabolite grouping information and excellent predictive ability of the model (Figure S3a). Further validation of the OPLS-DA model using the permutation test method showed that the R2′ and Q2′ of the replaced model were smaller than the original R2 and Q2, indicating the significance of the established OPLS-DA model (Figure S3b). Based on the OPLS-DA model, 50 DAMs were identified, of which 44 and 6 were up-accumulated and down-accumulated in the black chili peel, respectively (Figure 2a, Supplementary Table S3). These DAMs included flavonoids (52%), phenolic acids (14%), amino acids and derivatives (8%), alkaloids (8%), tannins (6%), lipids (4%), organic acids (4%), terpenoids (2%), and others (2%) (Figure 2b). Twenty-six flavonoids were up-accumulated in the black-skinned region, including 4 anthocyanins, 2 dihydroflavones, 9 flavonoids, 1 flavonoid carbonoside, and 10 flavonols (Figure 2c). Among them, the differentially accumulated anthocyanins included cyanidin-3*-O-*(6″-*p-*coumaroyl-glucoside), delphinidin-3*-O-*(6″-*p-*coumaroyl-glucoside), petunidin-3-(6″-*p-*coumaroyl-glucoside), and delphinidin-3-*p-*coumaroyl-rutinoid (Figure 2c).

### 3.3. Analysis of Differentially Expressed Genes Between the Two Different-Colored Pepper Fruit Peels

**Figure 3:**
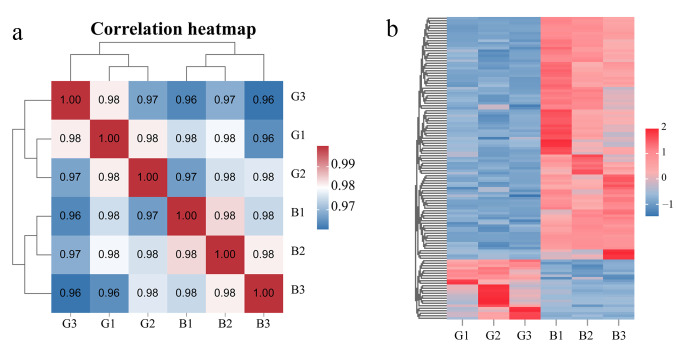
Correlation between gene expression levels and differentially expressed genes in different-colored pepper (*Capsicum annuum* L.) peels. (**a**) Correlation heatmap; (**b**) Differentially expressed genes heatmap. The heatmap displays Z-score-normalized FPKM values. B: black fruit peel, G: green fruit peel.

A total of 43.32 GB of raw data were obtained from six samples of fruit peels with two different colors. After filtering, the HQ clean data ratio of each sample was above 98.17%, and the Q30 of all samples was above 92.40%, indicating high sequencing quality (Supplementary Table S4). By comparing the genome and public databases, 28,798 genes were identified (Supplementary Table S4). Correlation analysis and clustering of the six samples showed that the correlation among the three biological replicates of the same color was higher than 97% and they clustered in one branch, indicating the reliability of the transcriptome sequencing results (Figure 3a). Based on the results of differential analysis, 121 DEGs were screened from black peel compared to green peel, of which, 97 DEGs were upregulated and 24 DEGs were downregulated in the black peel (Figure 3b, Supplementary Table S5).

### 3.4. Functional Analysis of DEGs of Two Different-Colored Peppers Fruit Peels

**Figure 4:**
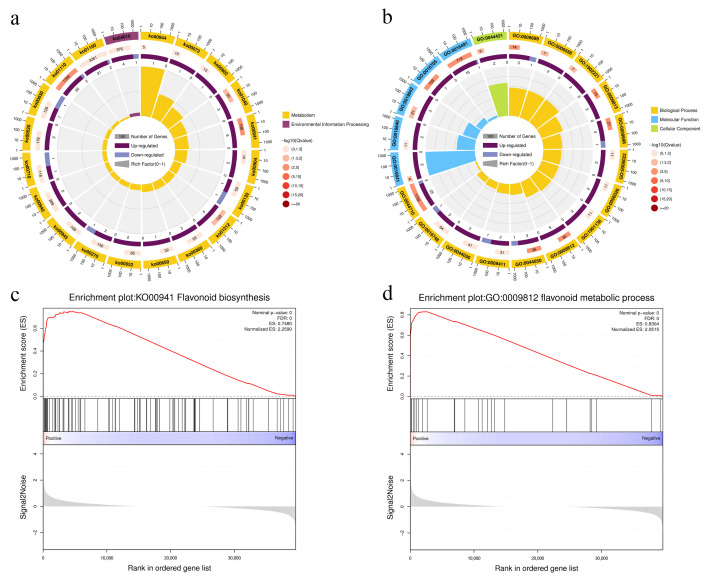
GO (**a**) and KEGG (**b**) enrichment analysis of differentially expressed genes of two different colored peppers (*Capsicum annuum* L.) peels. Gene set enrichment analysis (GSEA) of flavonoid biosynthesis (**c**) and flavonoid metabolic process (**d**).

A total of 44 DEGs were enriched in 46 pathways, including metabolic pathways (ko01100, 23 DEGs), biosynthesis of secondary metabolites (ko01110, 22 DEGs), flavonoid biosynthesis (ko00941, 6 DEGs), phenylpropanoid biosynthesis (ko00940, 5 DEGs), and MAPK signaling pathway plant (ko04016, 5 DEGs). Among these pathways, flavonoid biosynthesis (ko00941), biosynthesis of secondary metabolites (ko01110), biosynthesis of unsaturated fatty acids (ko01040), and fatty acid metabolism (ko01212) were significantly enriched (Figure 4a). In addition, based on the GO enrichment analysis, “extracellular region part” (GO:0044421) was enriched in the cellular components category; “oxidoreductase activity, acting on paired donors, with incorporation or reduction of molecular oxygen” (GO:0016705), “oxidoreductase activity” (GO:0016491), “ammonia-lyase activity” (GO:0016841), “vitamin binding” (GO:0019842) were enriched in the molecular function category; and “flavonoid metabolic process” (GO:0009812), “phenylpropanoid biosynthetic process” (GO:0009699), “phenylpropanoid metabolic process” (GO:0009698),”single-organism metabolic process” (GO:0044710), “flavonoid biosynthetic process” (GO:0009813), “secondary metabolite biosynthetic process” (GO:0044550), “L-phenylalanine metabolic process” (GO:0006558), and “erythrose 4-phosphate/phosphoenolpyruvate family amino acid metabolic process” (GO:1902221) were enriched in the biological processes category (Figure 4b). In addition, GSEA was used to analyze the expression trends of flavonoid metabolism genes between the two colored chili peels. The results showed that GSEA-KEGG and GSEA-GO were enriched in “flavonoid biosynthesis” (ko00941) and “flavonoid metabolic process” (GO:0009812) respectively (Figure 4c,d). These gene set members were also significantly upregulated. These transcriptome and metabolome results indicate that the observed differences in metabolites and gene expression affect anthocyanin biosynthesis in pepper peel.

### 3.5. Transcriptome and Metabolome Correlation Analysis in Anthocyanin Biosynthesis Process

**Figure 5:**
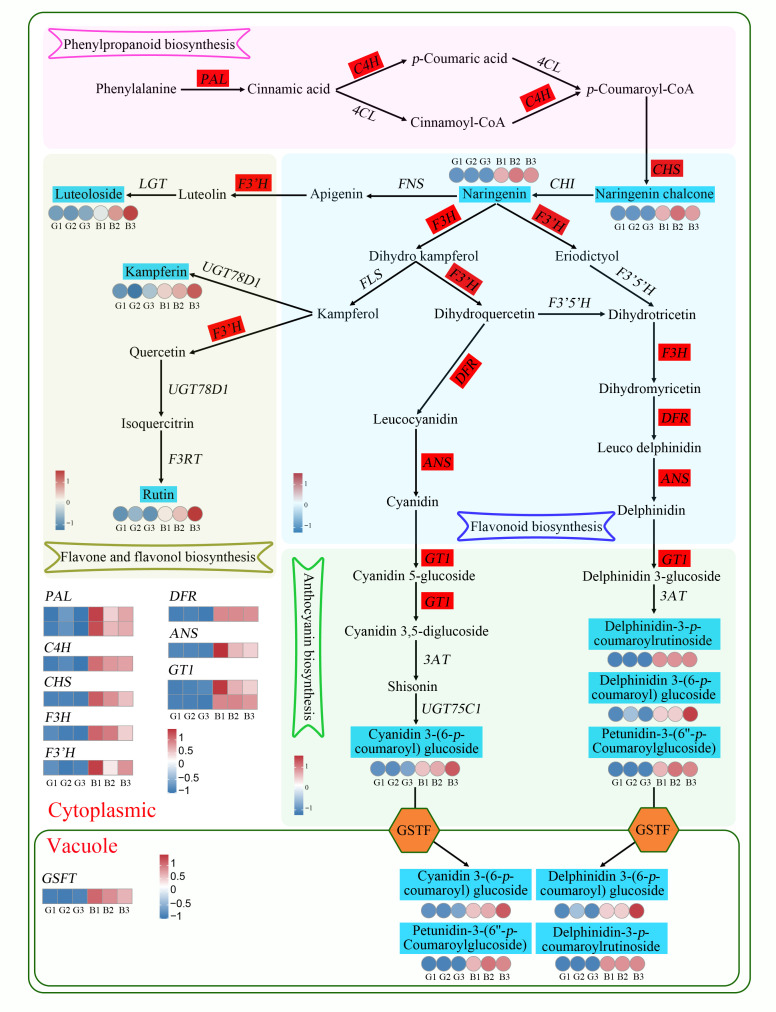
Metabolite content and gene expression characteristics of flavonoid and anthocyanin synthesis pathways in pepper (*Capsicum annuum* L.) peel. The circular heatmap represents differential accumulated metabolites, the box heatmap represents differential expressed genes. Metabolite content and FPKM values were normalized via Z-scores prior to heatmap generation. B: black fruit peel, G: green fruit peel.

Metabolome analysis revealed that flavonoids were differentially up-accumulated in black pepper peel and included three anthocyanins. Transcriptome analysis also found that DEGs were significantly enriched in “flavonoid biosynthesis,” indicating that the observed metabolites and DEGs were key factors affecting the biosynthesis of anthocyanin in pepper peel. Accordingly, we constructed a biosynthetic regulatory pathway for flavonoids and anthocyanins in pepper peel. In phenylpropanoid biosynthesis, two PAL genes (*Capana09g002200*, *Capana09g002190*) and one C4H gene (*Capana06g000273*) were upregulated in black fruit peels. In flavonoid biosynthesis, the metabolites naringenin (pme0376) and naringenin chalcone (pme2960) were upregulated by 122- and 180-fold in black fruit peels, respectively. The CHS (*Capana05g002274*), F3H (*Capana02g002586*), F3′H (*MSTRG.15987*), DFR (*Capana02g002763*), and ANS (*Capana01g000365*) genes were upregulated by 59-, 4-, 19-, 44-, and 40-fold in black fruit peels, respectively. In flavone and flavonol biosynthesis, the content of luteoloside (pme2459), kaempferin (mws0919), and rutin (mws0059) were upregulated by 5-, 2-, and 12-fold in black fruit peels, respectively. In the anthocyanin synthesis pathway, the contents of four anthocyanins, cyanidin-3*-O-*(6″-*p-*coumaroyl-glucoside) (*Lmpp003789*), delphinidin-3*-O-*(6″-*p-*coumaroyl-glucoside) (*Lmpp003662*), petunidin-3-(6″-*p-*coumaroyl-glucoside) (*Lmpp003815*), and delphinidin-3-*p-*coumaroyl-rutinoside (*Zmmp003591*) were upregulated by 5-, 8-, 19-, and 434637-fold in black fruit peels, respectively. The GT1 (*Capana03g000135*) gene, which is involved in anthocyanin synthesis, was upregulated by 7-fold in black fruit peels, and GSTF (C*apana02g002285*) gene, which is involved in anthocyanin transfer, was upregulated 53-fold in the black peel (Figure 5).

### 3.6. Correlation Analysis of Transcription Factors Related to Anthocyanin Synthesis with Differentially Accumulated Metabolites and Differentially Expressed Genes

**Figure 6:**
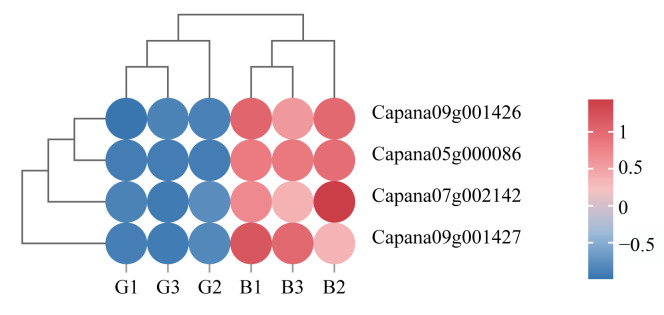
Heatmap of four differentially expressed transcription factors. The heatmap displays Z-score-normalized FPKM values. B: black fruit peel, G: green fruit peel.

The synthesis of secondary metabolites in plants is precisely regulated by transcription factors. Four transcription factors, including bHLH (*Capana09g001426, Capana09g001427*), TCP4 (*Capana07g002142*), and HSFB3 (*Capana05g000086*), were screened from the DEGs of the two different-colored pepper peels. All four transcription factors were upregulated in the black peel (Figure 6).

**Figure 7:**
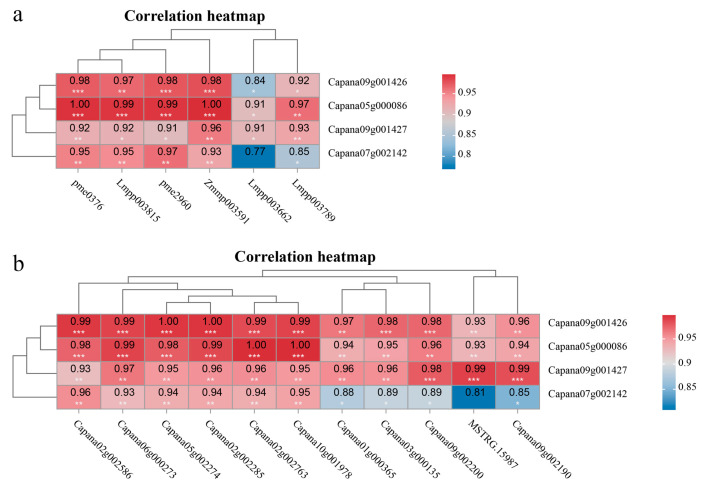
Correlation analysis of transcription factors with differentially accumulated metabolites (**a**) and key differentially expressed enzyme genes in anthocyanin synthesis (**b**). The numbers in the figure indicate the correlation coefficient, with * representing a significant correlation (*p* < 0.05), ** representing a highly significant correlation (*p* < 0.01), and *** representing an extremely significant correlation (*p* < 0.001).

To further investigate their potential regulatory roles in anthocyanin synthesis in pepper peel, a correlation analysis was conducted between these transcription factors and six DAMs involved in anthocyanin synthesis pathways. The results revealed that bHLH (*Capana09g001426, Capana09g001427*) and HSFB3 (*Capana05g000086*) exhibited significant positive correlations with all six differential metabolites (Figure 7a). Meanwhile, the transcription factor TCP4 (*Capana07g002142*) showed significant positive correlations with five specific metabolites: naringenin (pme0376), naringenin chalcone (*pme2960*), cyanidin-3*-O-*(6″-*p-*coumaroyl-glucoside) (*Lmpp003789*), petunidin-3-(6″-*p-*coumaroyl-glucoside) (*Lmpp003815*), and delphinidin-3-*p-*coumaroyl-rutinoside (*Zmmp003591*) (Figure 7a).

Further analysis of the correlations between the four transcription factors and key enzyme genes differentially expressed in the anthocyanin synthesis pathway revealed that bHLH (*Capana09g001426, Capana09g001427*) and HSFB3 (*Capana05g000086*) were significantly positively correlated with 11 DEGs (Figure 7b). Transcription factor TCP4 (*Capana07g002142*) was significantly positively correlated with 11 DEGs but not significantly correlated with F3′H (*MSTRG.15987*) (Figure 7b).

### 3.7. qRT-PCR

**Figure 8:**
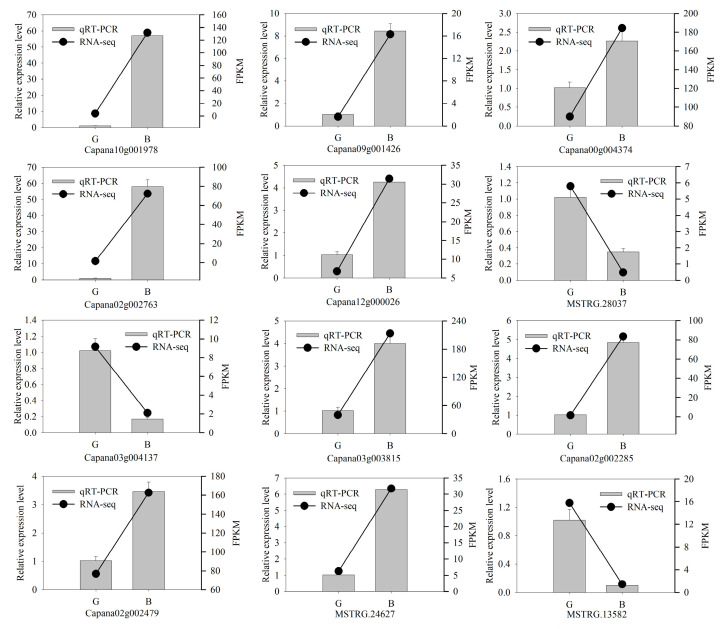
Quantitative reverse transcription PCR (qRT-PCR) and RNA-Seq data of 12 differentially expressed genes. B: black fruit peel, G: green fruit peel.

To further verify the accuracy and reproducibility of the transcriptome sequencing results, 12 DEGs were selected for qRT-PCR validation, including GT1 (*Capana03g000135*), bHLH (*Capana09g001426*), DFR (*Capana02g002763*), and GSTF (*Capana02g002285*) involved in anthocyanin synthesis. The qRT-PCR results of the 12 genes were consistent with the expression trend of transcriptome sequencing results, thereby indicating the reliability of the transcriptome sequencing results (Figure 8).

## 4. Discussion

Damage to plant cells caused by strong light and ultraviolet radiation is reduced by increasing the content of anthocyanins in plant tissues, thereby protecting plant growth [25]. Meanwhile, anthocyanins, as a natural food coloring, possess strong antioxidant and anti-inflammatory capacities and present obesity prevention and anti-cancer effects; thus, they are highly favored by consumers [29,30,31,32,33]. Therefore, the regulation mechanism of plant anthocyanin synthesis represents an important research topic. The biosynthesis of anthocyanins is influenced by many factors, with light representing one of the important factors [51,52,53]. In this study, the MSCJ1 variety of pepper fruit accumulated a large amount of anthocyanins in the illuminated area under natural light but did not form anthocyanins in the backlit area. Anthocyanin synthesis induced by light has been reported in other plants. Compared with white light, blue light can significantly increase the anthocyanin content of blueberry (*Vaccinium corymbosm*) fruits [54] and mixed red/blue light can also increase anthocyanin contents [55]. During the ripening process of non-climacteric bilberries (*Vaccinium myrtillus* L.), continuous exposure to red and blue light for 6 days resulted in 12-fold and 4-fold increase in anthocyanin concentration compared to natural light exposure, respectively [56]. Supplementing blue light at night can increase the content of anthocyanins in purple pepper fruits [57]. However, the metabolic basis and molecular regulation of the regional formation of anthocyanins in pepper fruits caused by light exposure remains unclear. In this study, a comprehensive analysis was conducted on the transcriptome and metabolome of anthocyanin biosynthesis in different-colored chili peels exposed to light. The findings provide a deeper understanding of the key metabolites, genes and metabolic pathways involved in anthocyanin biosynthesis and accumulation in pepper.

With the widespread application of metabolomics, the material basis of anthocyanin synthesis in different-colored peppers has been comprehensively studied [35,43]. Using extensive targeted and carotenoid targeted metabolomics analysis, a previous study identified differential metabolites of white, purple, green, and red fruit peels during the ripening process of chili peppers and revealed differences in 290 flavonoids and 68 carotenoids. Delphinidin-3*-O-*(2′′′*-O-p-*coumaroyl) rutinoside-5*-O-*glucoside and delphinidin-3*-O-*(2′′′*-O-p-*coumaroyl) rutinoside-7*-O-*glucoside were the main anthocyanins responsible for the formation of purple fruit peels [58]. Under blue light treatment, delphinidin-3*-O-*rhamnoside, delphinidin-3*-O-*rutinoside, and delphinidin-3*-O-*glucoside were highly accumulated in chili fruits, indicating that delphinidin glycosides are the main source of purple color [59]. In this study, a non-targeted metabolome analysis was used to identify 579 metabolites from two different-colored chili peels, of which 50 were differentially accumulated. These DAMs were mainly flavonoids (52%) and phenolic acids (14%), which are the material basis for the formation of black peel color. The DAMs involved in flavonoid metabolism and anthocyanin synthesis pathways in black chili peel included two flavonoids (naringenin and naringenin chalcone) and four anthocyanins [cyanidin-3*-O-*(6″-*p-*coumaroyl-glucoside), delphinidin-3*-O-*(6″-*p-*coumaroyl-glucoside), petunidin-3-(6″-*p-*coumaroyl-glucoside), and delphinidin-3-*p-*coumaroyl-rutinoid]. Naringenin and naringenin chalcone are upstream key substrates for anthocyanin synthesis. The contents of these two flavonoids was significantly higher in the black peel than the green peel and thus are key determinants of the color change of pepper black peel. These findings are consistent with results observed in eggplant [60]. Among the four anthocyanins, petunidin-3-(6″-*p-*coumaroyl-glucoside) and delphinidin-3-*p-*coumaroyl-rutinoid had the highest content in the black skin. Compared with the green skin, delphinidin-3-*p-*coumaroyl-rutinoid was 434,637.04-times higher in the black skin, indicating that petunidin-3-(6″-*p-*coumaroyl-glucoside) and delphinidin-3-*p-*coumaroyl-rutinoid are the main anthocyanins underlying black peel.

Anthocyanin synthesis involves pathways, such as phenylalanine metabolism, flavonoid biosynthesis, and anthocyanin synthesis. PAL is the rate-limiting enzyme in the phenylpropanoid pathway for anthocyanin synthesis. PAL gene expression and PAL enzyme activity are significantly positively correlated with anthocyanin synthesis [61,62,63]. C4H is a catalytic enzyme in the second step of the phenylpropanoid biosynthesis pathway, which can synthesize precursors of flavonoids [64]. In this study, PAL (*Capana09g002200*, *Capna09g002190*) and C4H (*Capana06g000273*) genes were up-regulated in black pepper peel, indicating that these two enzymes participate in anthocyanin regulation by initiating phenylalanine metabolism, leading to the formation of a black peel.

The flavonoid pathway can form anthocyanin substrates, and based on this synthesis pathway, anthocyanin synthesis genes can be divided into two categories: early structural genes (*CHS*, *CHI*, and *F3H*) and anthocyanin-specific biosynthesis genes (*F3′H*, *F3′5′H*, *DFR*, *ANS*, and *UFGT*) [60,65]. The *BpCHS2* gene in paper mulberry (*Broussonetia papyrifera*) exhibits a significant positive relationship with cyanidin derivatives. Overexpression of *BpCHS2* in tobacco (*Nicotiana tabacum*) significantly enhances anthocyanin accumulation [66]. Furthermore, overexpression of the VdCHS2 gene in spine grape (*Vitis davidii*) cells upregulates key downstream genes in the anthocyanin biosynthetic pathway-including *CHI*, *F3H*, *F3′5′H*, *DFR4*, and *LDOX*-ultimately leading to increased anthocyanin accumulation [67]. Transient overexpression of the blueberry *VcF3H2* gene in apple (*Malus domestica*) and blueberry (*Vaccinium* spp.) fruits upregulates the expression of structural genes involved in anthocyanin biosynthesis, thereby promoting anthocyanin accumulation in the fruit peel of both species [68]. Overexpression of the *FtF3*′*H1* gene in Arabidopsis, tobacco, and tartary buckwheat hairy roots can upregulate the expression of the *DFR*, *ANS*, and *FLS* genes involved in anthocyanin synthesis pathway, thereby promoting a significant increase in anthocyanin content [69]. Knocking out three genes (*OsF3*′*H*, *OsDFR*, and *OsLDOX*) in the anthocyanin biosynthesis pathway in black rice using CRISPR-Cas9 can significantly reduce the content of anthocyanins in black rice [70]. Genes, including *PAL, C4H, CHS, CHI, DFR, ANS,* and *UGT,* have also been shown to participate in the regulation of anthocyanin biosynthesis in pepper (*C. annuum*) fruits and promote anthocyanin accumulation in purple pepper [34,35,41]. In this study, CHS (*Capana05g002274*), F3H (*Capana02g002586*), F3′H (*MSTRG.15987*), DFR (*Capana02g002763*), ANS (*Capana01g000365*), and GT1 (*Capana03g000135*) were upregulated in the fruit peel of black pepper (*C. annuum*), indicating their involvement in anthocyanin biosynthesis in pepper pericarp.

The synthesized anthocyanins in the cytoplasm are unstable and prone to degradation. Plants typically transport anthocyanins to vacuoles for storage [71]. Proteins involved in anthocyanin transport primarily include MATE, ABC, and GST transporters [72]. Current studies have confirmed that GST participates in anthocyanin transport and accumulation across diverse plant species [73,74,75]. In upland cotton (*Gossypium hirsutum*) variety T586, *GhGSTF1* and *GhGSTF2* were highly expressed in red leaves and stems. Overexpression of *GhGSTF1* and *GhGSTF2* in the *Arabidopsis* mutant tt19-7 complemented the anthocyanin-deficient phenotype in hypocotyls [76]. In rose (*Rosa chinensis*), *RcGSTF2* overexpression significantly enhanced anthocyanin accumulation, while *RcGSTF1* silencing reduced anthocyanin content, demonstrating the critical involvement of *RcGSTF1* and *RcGSTF2* in regulating anthocyanin accumulation [77]. In this study, *GSTF* (*Capana02g002285*) was significantly upregulated in the black skin of pepper, indicating its regulation of anthocyanin transport from the cytoplasm to vacuoles in pepper peel cells.

The genes involved in plant anthocyanin synthesis are precisely regulated by transcription factors, including MYB, bHLH, WD40, MAD, and WRKY, which are involved in the regulation of anthocyanin biosynthesis [65]. Transient overexpression of *FabHLH110* in the petals of *Fragaria × ananassa* (pink-flowered strawberry) increased anthocyanin accumulation, while VIGS-mediated transient silencing of *FabHLH10* reduced anthocyanin content [78]. In *Malus halliana* (flowering crabapple), MhTCP4 promotes anthocyanin biosynthesis by directly activating the expression of *MhCHI* and *MhF3*′*H* in flowers [79]. In *Arabidopsis*, COP1/SPA complexes suppress anthocyanin biosynthesis by promoting the degradation of TCP3 in darkness [80]. In soybean, HSFB2b promotes flavonoid accumulation by activating the expression of genes related to flavonoid biosynthesis, thereby enhancing salt tolerance [81]. In this study, transcription factors bHLH (*Capana09g001426*, *Capana09g001427*), HSFB3 (*Capana05g000086*), and TCP4 (*Capana07g002142*) were upregulated in the black pericarps of pepper. Their expression showed significant positive correlations with most differentially accumulated anthocyanin metabolites and DEGs in the anthocyanin biosynthesis pathway, indicating that these four transcription factors participate in regulating anthocyanin synthesis in pepper.

However, this study only investigated the effects of light on metabolite and gene expression in pepper fruit as well as the anthocyanin metabolism pathways and key transcription factors. Moreover, how these transcription factors regulate the expression of key enzyme genes remains unclear. Further research will be conducted on the specific functions of these key enzyme genes and transcription factors to provide a deeper understanding of the regulatory mechanisms of anthocyanin biosynthesis and promote the breeding of new varieties of peppers rich in anthocyanins.

## 5. Conclusions

In this study, 579 metabolites were identified from the skin of black and green peppers, of which 50 were differentially accumulated. Key anthocyanin pathway DAMs included naringenin, naringenin chalcone, cyanidin-3*-O-*(6″-*p-*coumaroyl-glucoside), delphinidin-3*-O-*(6″-*p-*coumaroyl-glucoside), petunidin-3-(6″-*p-*coumaroyl-glucoside), and delphinidin-3-*p-*coumaroyl-rutinoside. Among these, petunidin-3-(6″-*p-*coumaroyl-glucoside) and delphinidin-3-*p-*coumaroyl-rutinoside were identified as the primary pigments contributing to black pericarp formation. RNA-seq analysis revealed 121 DEGs between the black and green pericarps. KEGG pathway analysis showed that these DEGs were significantly enriched in the flavonoid biosynthesis pathway. Critical anthocyanin biosynthesis enzyme genes and transporters and transcription factors regulating this pathway were identified. Our future research will focus on the regulation mode of transcription factors. The results presented herein provide novel insights into light-induced regulation of black pericarp formation in pepper and establish a theoretical basis for breeding anthocyanin-enriched pepper cultivars.

## Data Availability

The raw sequence data reported in this paper have been deposited in the China National Center for Bioinformation (https://ngdc.cncb.ac.cn/gsa, accessed on 14 August 2025) under accession numbers CRA029052. The data are presented in the article and Supplementary Materials.

## Supplementary Materials

The following supporting information can be downloaded at: https://www.mdpi.com/article/10.3390/cimb47090774/s1.

## Abbreviations

The following abbreviations are used in this manuscript

VIP	variable importance in projection
QC	quality control
ROS	reactive oxygen species
PAL	Phenylalanine ammonia lyase
C4H	cinnamic acid hydroxylase
4CL	coumarin CoA ligase
CHS	chalcone synthase
CHI	chalcone isomerase
F3H	flavonoid 3-hydroxylase,
F3′H	flavonoid 3′-hydroxylase
F3,5′H	flavonoid 3′-hydroxylase
DFR	dihydroflavonol 4-reductase
ANS	anthocyanin synthase
UGT (GT1)	UDP glucosyltransferase
GSTF	glutathione S-transferase
TIC	total ion chromatogram
GSEA	gene set enrichment analysis
OPLS-DA	Orthogonal partial least squares—discriminant analysis
PCA	PCA
