# Supplementary material for: Metabolome and Transcriptome Analyses of the Molecular Mechanism Underlying Light-Induced Anthocyanin Accumulation in Pepper (Capsicum annuum L.) Peel
Source: Curr Issues Mol Biol. 2025 Sep 18;47(9):774. doi: 10.3390/cimb47090774 (PMC12468451; doi:10.3390/cimb47090774)
Supplement: Supplementary file 1 [file cimb-47-00774-s001.zip › cimb-3859868-supplementary/Supplementary Figure.pdf]

current issues in molecular biology

MDPI

Article

Metabolome and transcriptome analyses of the molecular mechanism underlying light-induced anthocyanin accumulation in pepper (*Capsicum annuum* L.) peel

Qinqin He [1], Liming He [1], Zongqin Feng [1], Yunyi Xiao [1], Qiucheng Qiu [2], Jiefeng Liu [1], Hanbing Han [1] and Xinmin Huang [1,*]

Supplementary Figure

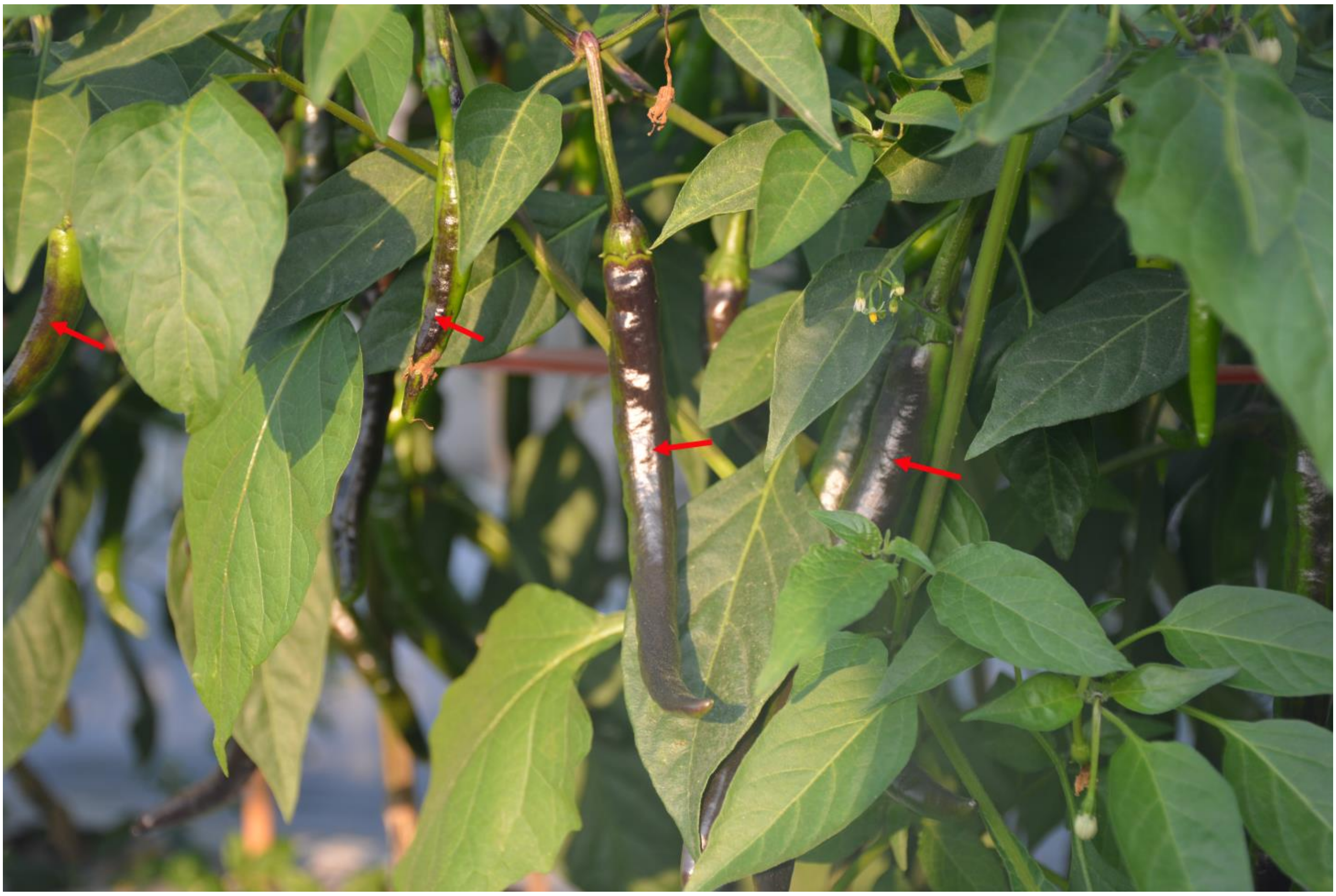

Figure S1. Field planting morphology of pepper (*Capsicum annuum* L.) MSCJ1. The red arrow represents the black peel area after illumination.

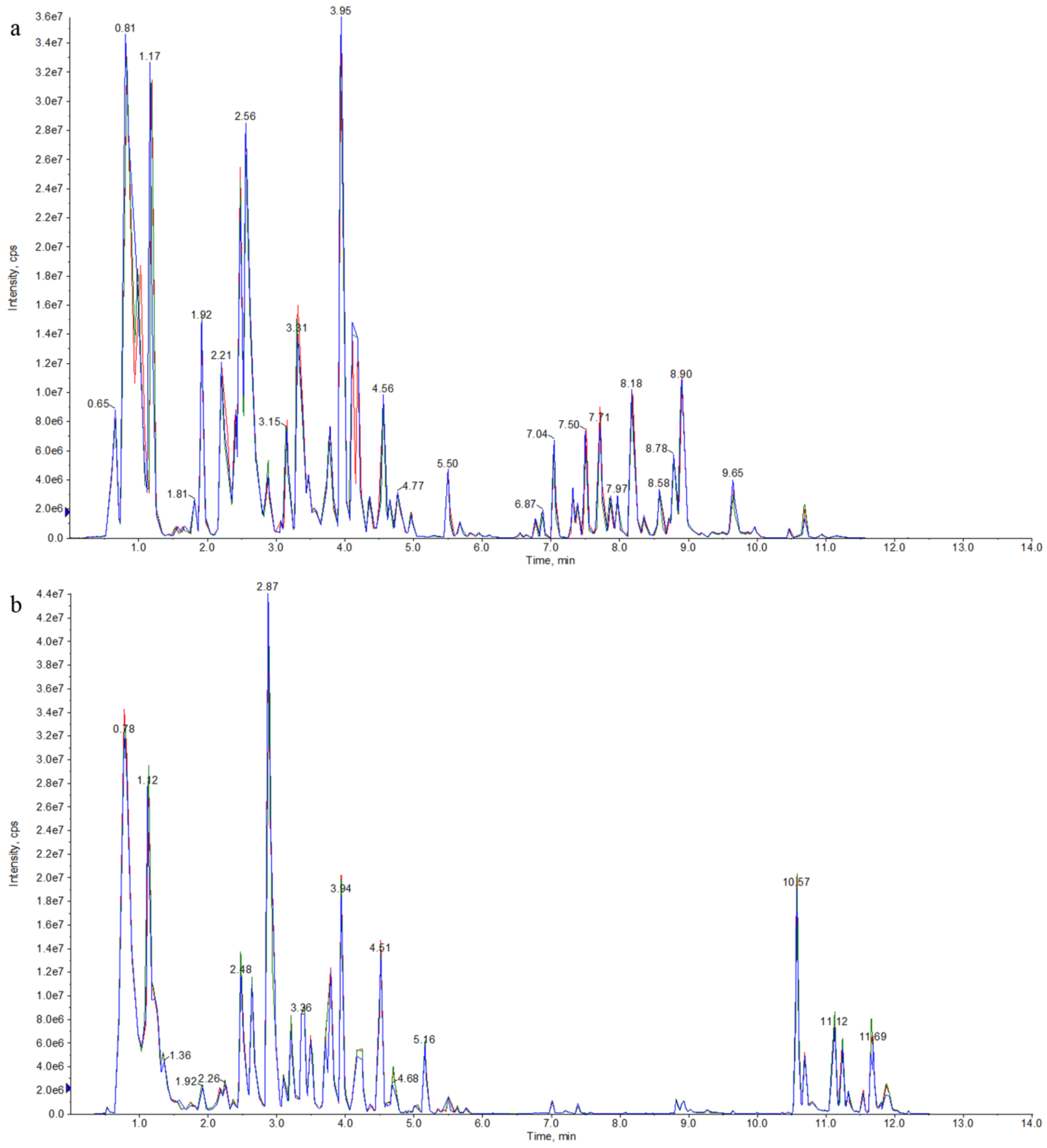

Figure S2. Total ion flow overlap diagram of quality control samples analyzed using mass spectrometry. a: positive ion; b: positive ion.

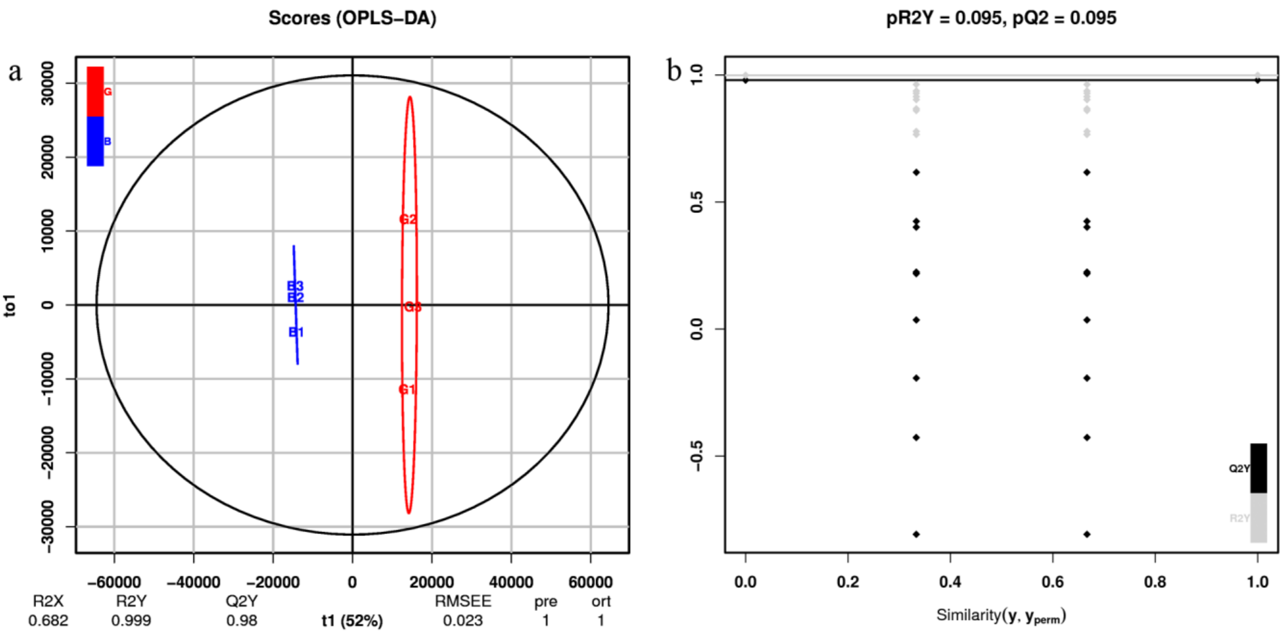

Figure S3. Orthogonal partial least squares–discriminant analysis (OPLS-DA) scores (a) and model validation plots (b) comparing metabolites from two different-colored pepper (*Capsicum annuum* L.) peels. B: black fruit peel, G: green fruit peel.
